# Supplementary material for: A web-database of mammalian morphology and a reanalysis of placental phylogeny
Source: BMC Evol Biol. 2007 Jul 3;7:108. doi: 10.1186/1471-2148-7-108 (PMC1941728; doi:10.1186/1471-2148-7-108)
Supplement: Additional File 1 — Website data: The 196 characters first described in [17], along with the supplementary data indicated below, have been joined into a number of HTML files, all of which are linked to the "morphsite_bmc07.html" index page. The embedded links to the supporting files are functional, independent of PC or platform, provided that the directory structure remains intact (i.e., all files remain in a single folder, no subdirectories). Nexus files: Datafiles containing the morphological, indel, and aligned DNA sequence data, linked from the "morphsite_bmc07.html" index page. Table 1. Changes to the multiple sequence alignment of [9]. Table 2. Qualitative character-state corrections to the morphology matrix of [17]. Table 3. Summary of discrepancies between Appendices 1 and 2 of [17]. [file 1471-2148-7-108-S1.zip › asher-BMC07-website/supp-tables.html]

## **Supplementary Table 1. Changes to the multiple sequence alignment of [9]. Only one change was made to non-protein coding sequences (APP), listed in the first row. Alignment changes are represented by smallcase nucleotides in nexus file available in the file "nuc-align.nex" , linked from the supplementary data website, the character positions of which correspond with locations given below in column 3. "AA" = amino acid; "RF" = reading frame.**

| **Gene** | **Taxa/on** | **Character(s) in nuc-align.nex** | **Change** |
| --- | --- | --- | --- |
| APP | all but *Cavia* | 1381 | single gap added to better align 19bp motif in *Cavia* |
| adrB2 | *Sorex* | 944-948 | indels in *Sorex* and *Equus* homologized |
| BDNF | *Trichechus* | 2973-2974 | gap characters changed to ? (no AA translation given in genBank AY011451) |
| BDNF | *Trichechus* | 3042 | gap character changed to ? (no AA translation given in genBank AY011451) |
| CNR1 | *Didelphis* | 3652-3654 | moved 1 position downstream to fit RF |
| TYR | *Equus* | 7948-8373 | *Equus* had not been included in Roca et al. (2004) alignment, sequences from AF252540 added |
| vWF | all | 8578-8592 | region reidentified as "alignment ambiguous" |
| vWF | ruminant | 9277-9282 | moved 1 position downstream to fit RF |
| vWF | *Didelphis* and *Macropus* | 9364-9366 | moved 3 bases downstream to match indel in *Sus* |
| BRCA1 | *Solenodon* | 9995-9997 | moved 1 position upstream to fit RF |
| BRCA1 | *Sorex* | 9998-10000 | moved 1 position upstream to fit RF |
| BRCA1 | *Solenodon* | 10046-10048 | moved 1 position downstream to fit RF |
| BRCA1 | phyllostomid | 10070-10084 | moved 1 position downstream to fit RF |
| BRCA1 | diprotodont | 10139-10141 | moved 1 position downstream to fit RF |
| BRCA1 | *Didelphis* and *Macropus* | 10712-10720 | moved 1 position downstream to fit RF |
| BRCA1 | all | 10730-10732 | moved 1 position downstream to fit RF |
| BRCA1 | *Echinops* | 10943-10945 | moved 3 positions downstream to match indel in other taxa |
| BRCA1 | *Didelphis* and *Macropus* | 11141-11149 | moved 2 positions downstream to fit RF and match indel in *Orycteropus* |
| BRCA1 | *Didelphis* and *Tupaia* | 11315-11317 | moved 6 positions upstream to match beginning of indel in cetartiodactyls |
| BRCA1 | *Chaetophractus* and myrmecophagid | 11315-11317 | moved 3 positions downstream to match beginning of indel in cetartiodactyls |
| BRCA1 | *Manis* | 11345-11356 | moved 3 positions upstream to match indel in marsupials |
| BRCA1 | *Orycteropus* | 11402-11404 | moved 3 positions downstream to match indel in *Manis* |
| BRCA1 | *Rousettus* | 11630-11641 | moved 2 positions upstream to fit RF |
| BRCA1 | *Tupaia* | 11891-11896 | moved 1 position upstream to fit RF |
| BRCA1 | *Erinaceus* | 12194-12205 | moved 1 position downstream to fit RF |
| BRCA1 | strepsirhine | 12536-12538 | moved 3 positions downstream to match indel in *Ochotona* and *Equus* |
| BRCA1 | phyllostomid | 12746-12748 | moved 3 positions downstream to fit indel in *Erinaceus* and *Lama* |
| BRCA1 | ruminant | 12830-12832 | moved 2 positions upstream to fit RF |
| IRBP | elephant shrews | 13944-13946 | moved 1 position downstream to fit RF |
| IRBP | *Didelphis* and *Ochotona* | 13950-13952 | moved 2 positions upstream to fit RF |
| IRBP | delphinid | 14055-14057 | moved 1 position downstream to fit RF |
| A2AB | *Orycteropus* and *Echinops* | 14799-14801 | moved 3 positions upstream to match termination of indel in bats, carnivorans, and *Manis* |
| A2AB | *Solenodon* and *Homo* | 14832-14834 | moved 1 position upstream to fit RF |
| A2AB | *Felis* | 15030-15032 | moved 2 positions upstream to fit RF |

**Supplementary
Table 2. Qualitative character-state corrections to the morphology
matrix first published in [17]. Asterisks indicate changes already
made by [24].**

| **character** | **taxon** | **as coded 2003** | **revised** |
| --- | --- | --- | --- |
| 11, Transpromontorial sulcus\* | *Ochotona* | internal carotid artery sulcus on ventrum of petrosal present (0) | sulcus absent (1) following Bugge (1974). |
| 20, Alisphenoid contribution to auditory bulla\* | *Zalambdalestes* | present (1) | absent (0) following Wible et al. (2004) |
| 33, Alisphenoid canal\* | *Leptictis* | polymorphic (0,1) | present (0) |
| 33, Alisphenoid canal\* | *Palaeoparadoxia* | present (0) | uncertain (?) |
| 39, hypoglossal foramen | *Orycteropus, Sus* | absent (0) | present (1) |
| 39, hypoglossal foramen | *Sorex* | absent (0) | polymorphic (0,1) |
| 48, Foramen rotundum | *Didelphis* | confluent (0) | distinct (1) following Wible (2003:153), Gregory (1910: 429) |
| 56, Glenoid position | *Manis* | glenoid poorly defined (2) | even with petrosal (0) |
| 71, Lacrimal foramen | *Sylvilagus* ("leporid" in present study) | with "fenestra in anterior orbit" comprising state 2 (state 2 also not recorded in Appendix 2B) | previous coding for *Sylvilagus* results from artefactual loss of lacrimal bone in some specimens. taxon "leporid" is corrected to state "0, lacrimal foramen present." |
| 75, Maxilla in medial orbital wall\* | *Amblysomus* | reduced (0), without incursion of maxilla in orbital wall | maxilla with incursion into medial orbital wall (1) |
| 178, Pedal digit I\* | *Ukhaatherium* | uncertain ("?") | weight bearing ("0") |

**Supplementary
Table
3. Summary of discrepancies between Appendices 1 and 2 of [17].**

| **Character (#, name)** | **Taxon** | **Correction** |
| --- | --- | --- |
| 25, ectotympanic shape | *Sorex* | Erroneously listed as state "1" in description on p. 173; should be state "0" |
| 41, petrosal exposure in braincase | *Tapirus* | State "0" (petrosal exposed) omitted from printed Appendix 1 |
| 43, dorsum subarcuate fossa | cetaceans | State 2 "absent" erroneously included in Appendix 2A. In matrix state "2" was coded as inapplicable (i.e., as missing) |
| 47, crista galli | All | States in Appendix 2B are reversed |
| 47, crista galli | cetaceans | Description for state 2 lacking in Appendix 2B |
| 57, glenoid shape | *Sorex* | Description of state 2 "glenoid dual" missing in Appendix 2B |
| 71, lacrimal foramen | All | States in Appendix 2B are reversed. |
| 76, premaxillary-frontal contact | cetaceans | Description of state 2 "present over maxilla" missing in Appendix 2B |
| 78, posterior nasals | *Arsinoitherium* | Description of state 2 "horns" missing in Appendix 2B |
| 89, palatal exposure premaxilla | bats, cetaceans, *Tamandua*, *Ceratotherium* | Description of state 0 "premaxilla absent or comprising alveoli only" missing in Appendix 2B; states 1 and 2 in matrix correspond to states 0 and 1 in Appendix 2B |
| 95, incisor enamel | *Loxodonta, Palaeoparadoxia* | Description of state 2 "enamel reduced, present on apex of tooth only" missing in Appendix 2B |
| 99, tooth replacement | marsupials | Following Luckett (1993), the last premolar of marsupial mammals, and the only tooth with a deciduous precursor, is p/P3, not p/P4 as originally written in Appendix 2B |
| 110, stylar shelf | All | States in Appendix 2B are reversed |
| 124, C2 odontoid shape | All | States 0,1 in Appendix 2B are reversed |
| 124, C2 odontoid shape | cetaceans | State 2 "odontoid missing" was erroneously included in Appendix 2B description for character 124, repeating information from character 123. However, state 2 was originally (and correctly) coded as inapplicable ("?") in matrix |
| 132, scapular shape | All | States in Appendix 2B are reversed |
| 132, scapular shape | talpids | Description of state 2 "scapula long and narrow" missing in Appendix 2B |
| 141, proximal radius | *Loxodonta* | Described in Appendix 2B as having state 2 "proximal radius articulates in V-shaped notch of ulna", but is coded in matrix as state 0 "proximal radius circular". Correctly coded as state 0 in matrix; with V-shaped notch accounted for in character 142. |
| 164, medial trochanteric ridge | *Didelphis* | Erroneously listed as state "1"; should be state "0" in description on p. 189 |
| 167, fibular facet of calcaneus | All | States in Appendix 2B are reversed |
| 177, projection from ankle | chiropterans | States in Appendix 2B for mega- and microchiropterans are reversed |
| 184, metatarsal length | *Macropus* | Erroneously given state 2 "long tibia"; should be coded state 1 for relatively long metatarsals |
